# Infant cannibalism in wild white‐faced capuchin monkeys

**DOI:** 10.1002/ece3.6901

**Published:** 2020-10-16

**Authors:** Mari Nishikawa, Nuria Ferrero, Saul Cheves, Ronald Lopez, Shoji Kawamura, Linda M. Fedigan, Amanda D. Melin, Katharine M. Jack

**Affiliations:** ^1^ Department of Integrated Biosciences Graduate School of Frontier Sciences The University of Tokyo Kashiwa Chiba Japan; ^2^ Área de Conservación Guanacaste Guanacaste Costa Rica; ^3^ Department of Anthropology & Archaeology University of Calgary Calgary AB Canada; ^4^ Department of Anthropology Tulane University New Orleans LA USA

**Keywords:** *Cebusimitator*, conspecific necrophagy, food sharing, infanticide, neophobia, thanatology

## Abstract

Cannibalism has been observed in a variety of animal taxa; however, it is relatively uncommon in primates. Thus, we rely heavily on case reports of this behavior to advance our understanding of the contexts under which it occurs. Here, we report the first observation of cannibalism in a group of wild white‐faced capuchin monkeys (*Cebus imitator*). The subject was a dead infant, estimated to be 10 days old, and the probable victim of infanticide. Consumption of the corpse was initiated by a 2‐year‐old male (second cousin of the infant), though it was eventually taken over and monopolized by the group's alpha female (grandaunt of the infant). Although most group members expressed interest in the corpse (sniffing, touching, and threatening it), no others made an attempt to consume it. Given that this is the only observation of cannibalism recorded in over 37 years of study on this population, we consider it to be a rare behavior in this species. This detailed record contributes new data, which, when combined with other reports within and across species and contexts, enables the evaluation of adaptive explanations of cannibalism.

## Graphical abstract

We report the first case of cannibalism observed in a group of wild white‐faced capuchin monkeys. The subject was a dead infant, estimated to be 10 days old. A 2‐year‐old male initiated the consumption of the corpse, though consumption was eventually taken over and monopolized by the alpha female.

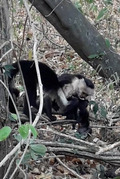

## 1 INTRODUCTION

Cannibalism, the consumption of all or part of another individual of the same species (Fox, 1975; Polis et al., 1984; Richardson et al., 2010), has been observed in a variety of animal taxa (Elgar & Crespi, 1992; Fouilloux et al., 2019; Polis, 1981). Also known as *conspecific necrophagy* (Anderson, 2011), the consumption of conspecifics may be adaptive under some circumstances as it provides access to high‐quality proteins, lipids, micronutrients, vitamins, and minerals (Milton, 2003; Snyder, 2000). However, this practice also has associated risks, such as pathogen transmission (Lindenbaum, 1979; Pfennig et al., 1998; Rudolf & Antonovics, 2007), possible minor or fatal injuries (Polis, 1981), and decreased inclusive fitness if the victim is a relative (Fea et al., 2014; Pfennig, 1997).

Cannibalism has been observed to occur under a wide array of contexts linked to variation in ecological and/or social conditions (Schutt, 2017). For example, environmental stressors, such as food scarcity and high population density, can lead to cannibalistic behavior in arachnids and cephalopods (Ibáñez & Keyl, 2010; Mayntz & Toft, 2006) and sexual cannibalism (the consumption of male partners by females during courtship or mating) is widespread in arachnids and in several insect orders (Buskirk et al., 1984; Elgar, 1992; Wilder et al., 2009). In mammals, cannibalism is commonly associated with infanticide (Polis et al., 1984); in primates, about half of the reported cases of infant cannibalism involved infanticide, while maternal cannibalism of deceased infants is the next most common context (Tian et al., 2016).

Here, we report an incident in which a wild infant white‐faced capuchin (*Cebus imitator*) was cannibalized by group members at our study site in northwestern Costa Rica. Detailed accounts of cannibalism in Central and South American primates are exceptionally rare with only eight cases observed in six different species (*Callithrix jacchus*: Lazaro‐Perea et al. (2000), Melo et al. (2003), Bezerra et al. (2007); *Callithrix flaviceps*: Hilário and Ferrari (2010); *Saguinus fuscicollis*: Herrera et al. (2000); *Saguinus mystax*: Culot et al. (2011); *Saimiri sciureus*: Manocha and Long (1977); and *Sapajus apella*: Trapanese et al. (2020)). Among capuchins, to date, only a single incident of cannibalism has been reported (captive *Sapajus apella*: Trapanese et al. (2020)). Our observation adds important information about this taxonomically widespread, though rare and poorly understood, behavior and will thus advance the understanding of the contexts under which this behavior occurs. In addition, the events described herein will add to the growing field of comparative thanatology, the scientific study of death, which includes the analysis of death awareness and empathy (Anderson, 2011, 2016).

## 2 METHODS

On 9 April 2019, we observed a case of cannibalism in the LV group of capuchins in the Santa Rosa Sector of the Área de Conservación Guanacaste, Costa Rica. During the event, we collected data ad libitum and video data using two cell phones. Long‐term research on the Santa Rosa capuchins began in 1983 (Fedigan & Jack, 2012; Melin et al., in press) with observations of the LV group beginning in 1990. All group members were fully habituated and individually identifiable based upon physical features. We present key individuals and relationships in Table 1. The family pedigree of this group is shown in Appendix S1, and a detailed report with time stamps of this event is presented in Appendix S2.

**Table 1:** Key individuals involved in the cannibalism

Monkey ID	Sex and age	Relationship to cannibalized infant
CT	Primiparous adult female aged 7 years	Mother of CT‐19
CT‐19	CT’s first infant (d.o.b. estimated to within 4 days: 3/29/2019)	Self
SS	Alpha female aged 23	Aunt of CT
BS	Juvenile male aged 2 (d.o.b 2/2017)	Grandson of SS, second cousin of CT‐19
MC	Juvenile male aged 2 (d.o.b 1/2017)	Son of SS
PW	Subordinate adult male aged 13, immigrated in 2016	Unknown

## 3 RESULTS

### 3.1 Prior to cannibalism

CT, an adult female, was the subject of a focal animal sampling session from 12:01 to 14:01, during which her infant CT‐19 was always clinging and appeared healthy. Prior to the start of sampling (around 11:30), CT, CT‐19, and SS (the alpha female and grandaunt of CT‐19) were resting together in a tree. At 12:36, an agonistic encounter between study group LV and the neighboring RM group occurred. CT moved away from the conflict and rested in a tree, ca 5 m high, near a juvenile and an adult male. We were unable to identify these two nearby individuals as their faces were blocked by vegetation. CT continued to rest, nurse CT‐19, and groom the juvenile and the male until the focal session ended at 14:01, when we finished watching CT.

At 14:03, we heard loud vocalizations (“screams”; Gros‐Louis et al., 2008) from the direction of the branch where CT was just observed. Soon after, CT‐19 fell to the ground from a tree. CT rushed down to the ground and approached CT‐19 who grabbed CT’s belly with her hands, but CT‐19’s back legs were not functioning. Immediately following the scream vocalizations, we observed an adult male PW being chased from the area by an adult female. CT retreated to the tree with CT‐19, but CT‐19 fell to the ground. CT again retrieved CT‐19 and moved to the tree, but again CT‐19 fell from ca. 4 m high. After the third fall, we noticed blood on CT‐19’s side around the lateral region of the abdomen. CT‐19 could no longer hold on to her mother. CT then directed alarm calls (Gros‐Louis et al., 2008) and double threats (one monkey jumps on the back of another and aligns its head with that of its ally to threaten an opponent: Fedigan (1993)) with three adult females toward CT‐19 who was quiet and motionless on the ground. Other individuals approached and showed interest in the infant (touching, gazing, licking, and sniffing). At 14:24, CT‐19 was presumed dead, as observers no longer detected movement or breathing. PW returned 10 min later and looked at CT‐19. CT approached and directed a threat face (Perry, 1996) at PW, but he did not display any reaction.

### 3.2 During cannibalism

**Figure 1:**
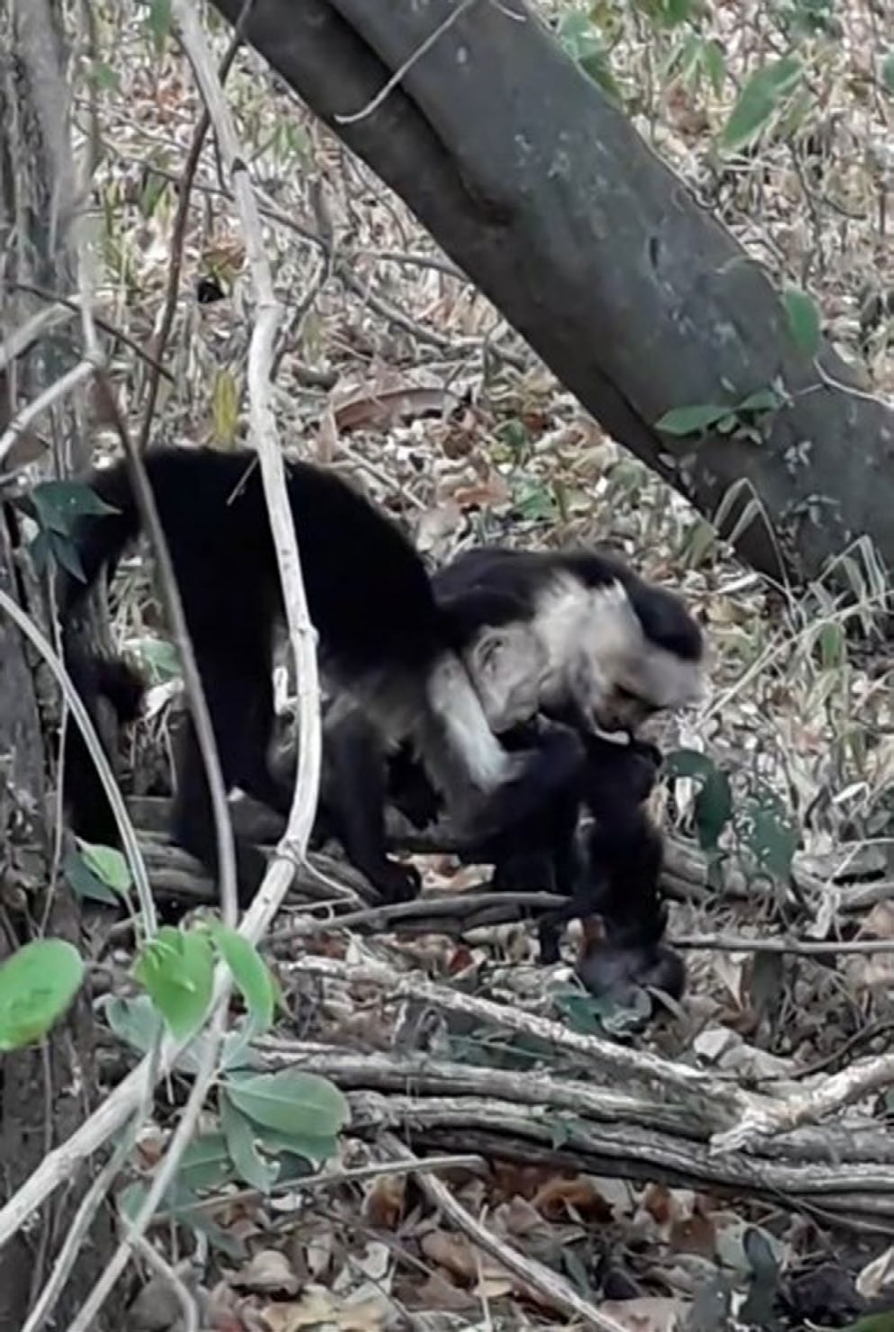
Adult female (right, SS) and juvenile male (left, BS) holding and eating the dead infant (CT‐19) (Photograph by N. Ferrero)

**Figure 2:**
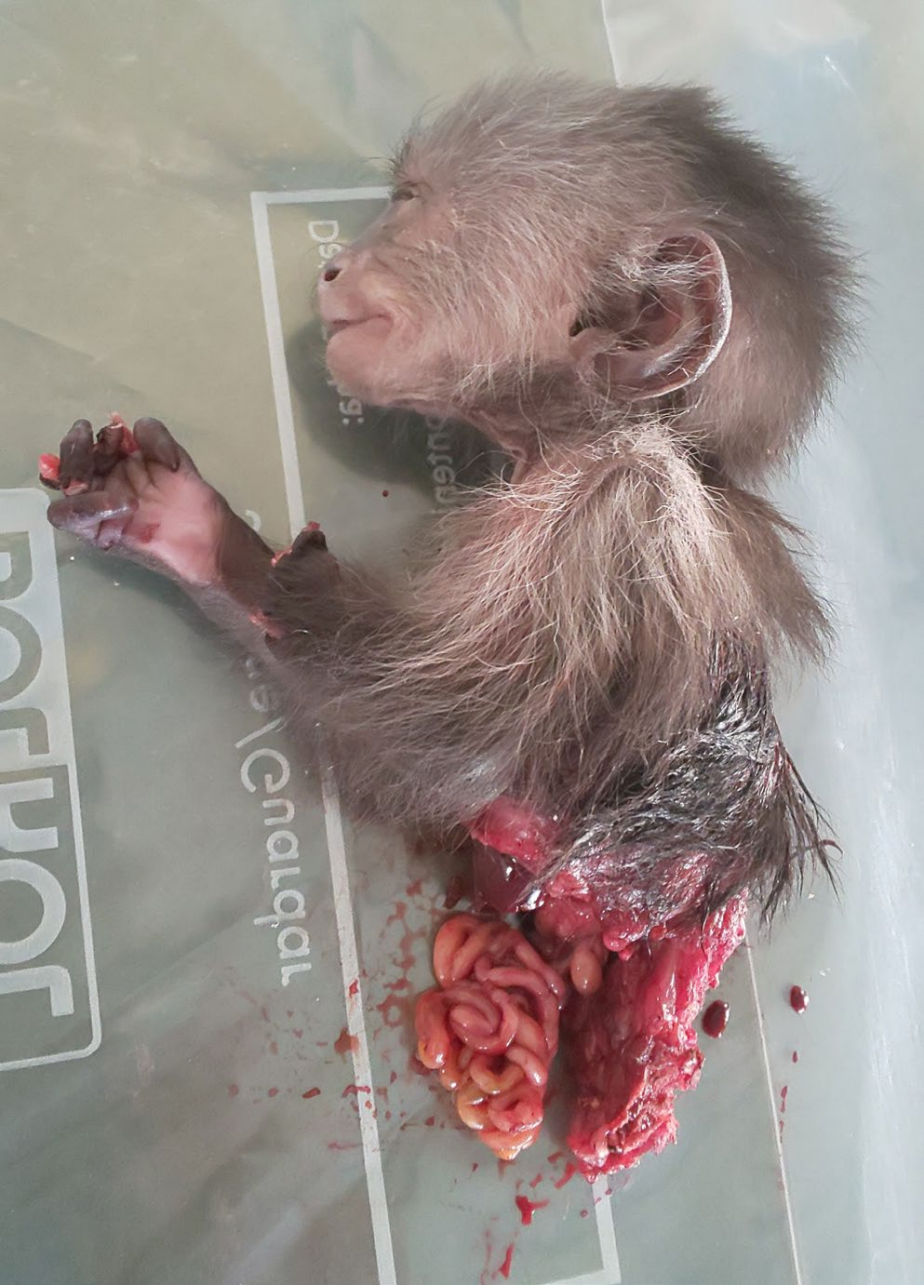
Remains of infant corpse following observed case of cannibalism in white‐faced capuchins (Photograph by M. Nishikawa)

In brief, at 14:39, we observed a juvenile male BS (grandson of SS) with the CT‐19’s corpse on the ground and he bit off and began to eat the toes of CT‐19’s left foot. SS, who was near, approached BS and licked the corpse. BS carried the corpse up into a tree and continued to consume it. Though CT made no attempt to retrieve the corpse, she remained close by and vigilant, watching BS and the corpse. At 14:53, BS dropped the corpse to the ground. Soon after, several individuals gathered around the corpse and visually inspected, sniffed, and touched the corpse. BS climbed down to the corpse and continued to eat the left leg of the corpse. Shortly after, SS, who was still in proximity and touching the corpse, pulled the corpse away from BS with no obvious aggression, and SS started to eat the corpse's left foot. Immediately thereafter, BS bit off a piece of the corpse's tail and began to eat it (Figure 1). SS eventually mildly threatened BS, who screamed submissively and climbed into a tree with the piece of tail (Video S1). SS consumed the corpse for 31 min, while BS and another juvenile male (MC, 2‐year‐old son of SS) remained within 2 m, watching her eat and sometimes touching the corpse. At 15:40, SS discarded the corpse after having eaten the remaining parts of the lower body, leaving its head, chest, and arms (Figure 2).

### 3.3 After cannibalism

The discarded corpse attracted attention and investigation from two adult males and adult females, including the mother, who approached and emitted vocalizations (alarm calls and trills; Gros‐Louis et al., 2008) and facial threats toward the corpse (Video S2). At 15:57, all group members left from the area, moving away to forage. After 15 min during which none of the monkeys returned, we collected the remains of the corpse for photographs.

## 4 DISCUSSION

This is the only case of cannibalism reported in the Sector Santa Rosa study population in over 37 years of observation on 5 + study groups. Additionally, there are no published reports of this behavior from the nearby site of Lomas Barbudal, where capuchins have been studied since 1990 (Perry et al., 2012).

The cannibalized infant, CT‐19, was estimated to be 10 days old at time of death. Immediately following the screams and the infant's fall to the ground, adult male PW was chased from the same area by an adult female. Prior observations of infanticide in this species have documented similar reactions by group females following infanticidal events (e.g., Perry & Manson, 2008). Although we did not directly observe the event leading to the infant's fall from the tree and the cause of infant's injury, the situation supports the assumption that the mother and the infant were involved in an aggressive exchange with PW. Hence, we suspect CT‐19 might be a victim of infanticide by PW.

Following the death of their young infants, female capuchins usually carry them for many hours (e.g., Schoof et al., 2014), a behavior that is even common for primiparous females (Perry & Manson, 2008). In the case we report here, although the mother presented herself to the infant, she made no attempt to carry it when it was unable to cling to her. A possible proximate explanation for the cannibalism is that, once the infant could no longer cling, the mother made no effort to retrieve its dead body or restrict access of other group members to it. This might have been due, in part, to her inexperience handling infants.

*Cebus imitator* are omnivorous and often eat small vertebrates including lizards, squirrels, birds, and nestling coatis (Fedigan, 1990; Rose, 1997). When capuchins catch vertebrate prey, agonism for access to the prey often arises among individuals (Fedigan, 1990), food sharing occasionally occurs, and the entire animal is consumed. In this observed case of cannibalism, however, only an alpha female and a juvenile consumed the meat, whereas other group members merely inspected or threatened the corpse. Because unfamiliar objects, foods, and situations elicit a neophobic or neophilic reaction, or a mixture of both (Russell, 1973), we suggest that this observed case was an unusual situation for the capuchins. The juvenile's behavior might have stimulated the alpha female to consume the infant because social information reduces neophobia (Forss et al., 2017). Additionally, when capuchins capture prey, they usually bite the face, possibly to avoid being bitten or to silence the prey. In the case described here, the CT‐19’s face was not bitten and much of the upper body remained relatively untouched (Figure 2). The alpha female discarded the uneaten head, upper limbs, and torso of the corpse, and no other group members attempted to consume it. Thus, the dead infant was not treated like other prey species. This pattern of not consuming the entire corpse is similar to reports of cannibalism following infanticide in chimpanzees (Kirchhoff et al., 2018).

To date, most cases of infant cannibalism in primates have followed infanticide by unrelated adult individuals or the corpses are consumed by closely related individuals of the infant after its natural death, for example, mother or sibling (Tian et al., 2016; Trapanese et al., 2020). In this case, the initiator of cannibalism was a 2‐year‐old juvenile, who was not involved in the CT‐19’s death and distant relative to her. Novel behaviors being instigated by juveniles have been reported in numerous primate species and might have played a role in this new behavior (Cambefort, 1981; Hannah & McGrew, 1987; Kawai, 1965; Perry, 2020). However, the significance of the initiator being a juvenile is unclear given we have only one data point, and other explanations are possible (Trapanese et al., 2020).

The nutritional hypothesis for cannibalism posits that the act of consuming a conspecific provides participating individuals with nutritional benefits that might improve future reproductive success (Fox, 1975; Klug & Bonsall, 2007). While we are unable to test this hypothesis directly, several factors provide some general support. First, the alpha female gave birth 13 days after the cannibalism event; thus, she was in an advanced state of pregnancy at the time. In mammals, pregnancy increases daily energy expenditure by 20%–30% (Aiello & Wells, 2002). Therefore, the alpha female might have been motivated by higher than average hunger and was able to gain unchallenged access to the corpse via her high dominance status. In addition, BS’s mother had recently given birth, indicating that he was recently weaned, so he may have been seeking supplemental nutrition. Future meta‐analyses of accumulating reports may be able to test nutritional hypotheses about this rare behavior. Our report contributes to this goal by providing a detailed account of a first‐time observation from a wild and unprovisioned population of capuchins.

## CONFLICT OF INTEREST

None declared.

## AUTHOR CONTRIBUTION

**Mari Nishikawa:** Conceptualization (lead); Data curation (lead); Formal analysis (lead); Funding acquisition (lead); Investigation (lead); Methodology (lead); Resources (lead); Software (lead); Visualization (lead); Writing‐original draft (lead); Writing‐review & editing (lead). **Nuria Ferrero:** Investigation (supporting). **Saul Cheves:** Investigation (supporting). **Ronald Lopez:** Investigation (supporting). **Shoji Kawamura:** Supervision (supporting); Writing‐review & editing (supporting). **Linda M Fedigan:** Data curation (supporting); Project administration (equal); Supervision (supporting); Writing‐review & editing (supporting). **Amanda Dawn Melin:** Data curation (supporting); Methodology (supporting); Project administration (equal); Supervision (supporting); Writing‐review & editing (equal). **Katharine M Jack:** Conceptualization (supporting); Data curation (supporting); Investigation (supporting); Methodology (supporting); Project administration (equal); Resources (equal); Supervision (supporting); Visualization (supporting); Writing‐original draft (supporting); Writing‐review & editing (equal).

## Supporting information

Appendix S1

Appendix S2

Video S1

Video S2

## Data Availability

A detailed report with time stamps of this event will be archived in Dryad on manuscript publication (https://doi.org/10.5061/dryad.rbnzs7h9g).
