# Supplementary material for: Infant cannibalism in wild white‐faced capuchin monkeys
Source: Ecol Evol. 2020 Oct 16;10(23):12679–84. doi: 10.1002/ece3.6901 (PMC7713969; doi:10.1002/ece3.6901)

**Article title**: Infant cannibalism in wild white-faced capuchin monkeys
**Journal name**: Ecology and Evolution
**Author names**: Mari Nishikawa, Nuria Ferrero, Saul Cheves, Ronald Lopez, Shoji Kawamura, Linda M Fedigan, Amanda D Melin, Kathy M Jack
**Corresponding author**: Mari Nishikawa, Department of Integrated Biosciences, Graduate School of Frontier Sciences, The University of Tokyo, nishikawa.mari@edu.k.u-tokyo.ac.jp

**APPENDIX 1**. Family pedigree for individuals involved in the cannibalism event. Note that adult males were immigrants to the group and the all adult males immigrated from neighboring groups in 2016.

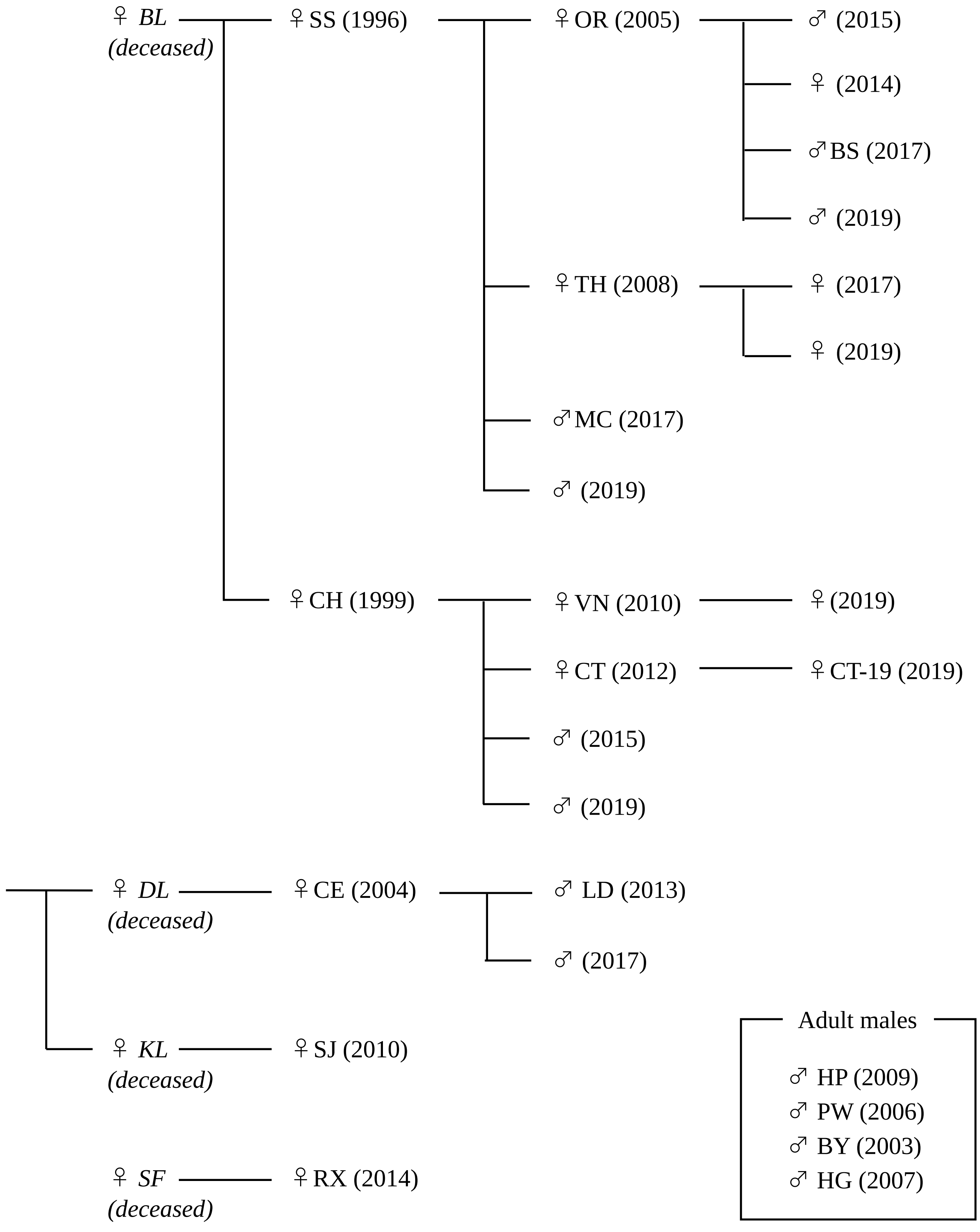

Supplement: Supplementary file 1 — Appendix S1 [file ECE3-10-12679-s001.pdf]
