# Supplementary material for: Infant cannibalism in wild white‐faced capuchin monkeys
Source: Ecol Evol. 2020 Oct 16;10(23):12679–84. doi: 10.1002/ece3.6901 (PMC7713969; doi:10.1002/ece3.6901)
Supplement: Supplementary file 2 — Appendix S2 [file ECE3-10-12679-s002.docx]

**Article title**: Infant cannibalism in wild white-faced capuchin monkeys

**Journal name**: Ecology and Evolution

**Author names**: Mari Nishikawa, Nuria Ferrero, Saul Cheves, Ronald Lopez, Shoji Kawamura, Linda M Fedigan, Amanda D Melin, Kathy M Jack

**Corresponding author**: Mari Nishikawa, Department of Integrated Biosciences, Graduate School of Frontier Sciences, The University of Tokyo, nishikawa.mari@edu.k.u-tokyo.ac.jp

**APPENDIX 2**

Detailed chronological description of the cannibalism event (April 9, 2019). See Appendix 1 for information on individual group members and their kinship.

CT was an adult female and she was the focus of a 120 min focal animal sampling (Altmann, 1974) session by Nishikawa and an assistant from 12:01 – 14:01.

**11:30** – CT, CT-19 (infant of CT), and adult alpha female SS (grandaunt of CT-19) were resting together in a *Ficus* tree at the campground area.

**12:01** – CT traveled to a feeding patch of *Acrocomia aculeate* and ate a fruit.

**12:13** – CT finished feeding, and she nursed CT-19 and rested in a tree at the campground. Most of other group members also rested together near a water tank in this vicinity.

**12:36** – A neighboring capuchin group, RM, entered the campground area. LV group left the campground and moved away from the water tank ca. 80 m north, and gathered in a few trees apart and rested.

**12:41** – CT nursed CT-19.

**12:52** – The adult males of LV group, including alpha male HP, went back to the campground and they had aggressive interaction with RM group.

**13:05** – HP rejoined adult females and juveniles of LV group. CT rested with a juvenile and adult male in close proximity in a tree, ca 5 m high, and nursed CT-19. CT continued resting and exchanged grooming and resting in contact with the two individuals in the same branch of the tree. Most of the members in LV group rested within 10 m of CT throughout the focal session.

**14:01** – The observers finished focal animal sampling of CT.

**14:03** – The observers heard loud vocalizations, “screams”, of capuchins from the direction of the branch where CT has just been observed. Soon after, CT-19 fell to the ground from a tree. The observers moved away ca. 5 m so as not to disturb capuchins’ behavior. Although the observers could not see the direct interaction surrounding CT-19 at that moment, immediately after hearing the capuchin screams, the observers saw adult male PW, being chased from the area by an adult female. CT immediately descended and approached CT-19. CT-19 grabbed CT’s belly with her hands but CT-19’s back legs appeared to not be functioning. CT ascended a tree with CT-19 hanging from her. She made no effort to hold the baby to her.

**14:09** – CT-19 fell to the ground again and CT immediately came down to the ground and approached her infant. CT-19 grabbed CT’s belly with her hands and CT ascended a tree again with CT-19 hanging from her, but soon after CT-19 fell to the ground. CT descended to the ground again and pressed her belly to CT-19, but CT-19 did not grab on though she was still moving. CT did not make attempts to carry CT-19 with her hands. Then, the observers noticed blood on CT-19’s one side of rib cage. CT stayed around CT-19 and pressed her belly towards the infant many times and touched the body. At this time some group members started to gather around CT-19.

**14:13** – CT emitted an alarm call and held CT-19’s body. Soon after, subadult female RX descended from a tree near CT and approached CT-19. RX touched CT-19 and licked its trunk. After that, adult females OR and CE, and alpha adult male HP also approached CT-19.

**14:19** – CT and CE directed a double threat toward CT-19. CT approached CT-19 again and touched it.

**14:21** – Adult female SJ and subadult male LD approached CT-19.

**14:23** – CT and SJ exhibited a double threat toward CT-19, after that CT approached CT-19 and licked its fur of trunk.

**14:24** – CT-19 was presumed dead, as observers no longer detected movement or breathing.

**14:26** – CT emitted alarm calls toward CT-19.

**14:31** – Adult female CH (grandmother of CT-19) approached CT-19 and watched it in close proximity.

**14:32** – CT and adult female TH exhibited a double threat toward CT-19, and TH approached CT-19 and touched and sniffed it.

**14:34** – PW returned and looked at CT-19, and CT approached PW and sat 1 m away from him.

**14:36** – CH touched CT-19.

**14:37** – CT directed a threat face towards PW, but he did not show any reaction. CT ascended a tree, leaving CT-19 on the ground. After which many group members approached CT-19 and touched and sniffed it. CT watched from the tree.

**14:38** – A juvenile touched CT-19 and moved it and CH and SS also touched it. Then, CT descended to the ground from tree and approached CT-19.

**14:39** – Juvenile male BS started to bite off and eat a piece of CT-19’s left toe. SS approached and licked CT-19’s right hand, and BS continued eating the piece of foot sitting next to SS. Adult female VN was looking at them from a tree 1 m away, and two juveniles also observed from a tree within 2 m. There were at least another 9 individuals within 10 m from CT-19.

**14:41** – BS ascended a tree with the piece of foot and continued to consume it. Soon after, a juvenile approached BS and looked closely at the piece. Then, the juvenile touched the piece held by BS and then licked the finger that touched the foot.

**14:43** – The juvenile left BS.

**14:52** – BS descended the tree and took whole body of CT-19 from the ground back up the tree. CT followed BS into the tree and approached to within 0.5 m and observed which BS started to hurriedly consume CT-19’s left hand. Soon after, CT left BS. VN and a juvenile approached BS and looked at him. BS tried to keep a distance from them while still holding CT-19. BS continued to hurriedly eat CT-19. VN and the juvenile approached BS again and directed a double threat toward CT-19. HP and another juvenile also approached BS and directed threat faces to CT-19.

**14:53** – BS jumped off but he dropped CT-19 on the ground. Soon after, HP, SS, OR, BS, and juvenile male MC gathered around CT-19 and closely visually inspected the infant. BS approached and took a bite of CT-19 again. OR and MC touched CT-19 and OR licked it. SS held CT-19’s left arm and looked at it. Soon after, BS started to bite and eat CT-19’s left leg. MC touched CT-19’s left arm and OR lifted the right arm and licked it. Soon after, MC and OR left. BS continued eating CT-19 and SS still kept holding its hand.

**14:54** – SS pulled CT-19 away from BS, but he resisted and resumed eating the left arm. SS pulled the infant again from BS and then started to eat CT-19’s left arm herself. BS stayed near by SS and touched CT-19. SS did not show any aggressive behavior towards BS.

**14:55** – CH approached SS and gazed at CT-19 in close proximity, but she left shortly after. SS continued eating CT-19.

**14:56** – A juvenile approached CT-19 and touched and sniffed it.

**14:57** – SS started to eat CT-19’s left foot and BS bit off a piece of CT-19’s tail at the same time while sitting next to SS (Figure 1, Appendix 3). Another juvenile stayed around them in close proximity and watched.

**14:58** – SS threaten BS and he emitted screams and ascended a tree ~ 3m away, while still holding the piece of tail. SS ascended a tree and continued holding and eating the body of CT-19, with BS and MC watching SS. Those two juveniles stared into CT-19 held by SS and sometimes touched it. SS did not direct any aggression towards the two juveniles. Note that MC was SS’s 2-year-old son and BS was her 2-year-old grandson.

**15:22** – BS ate a piece of CT-19 held by SS.

**15:25** – SS discarded a piece of CT-19’s skin with fur, and then BS descended to the ground and picked up and ate it.

**15:40** – SS discarded CT-19 after she ate its lower body, leaving the head, chest, and forelegs, though several fingers had been consumed (Figure 2). Adult males HG and BY, came to see CT-19 soon after SS discarded it, but they kept a distance ca. 2 m from CT-19 and directed threat faces to CT-19.

**15:44** – HG and BY exhibited a double threat toward CT-19. SS also exhibited a double threat with a juvenile toward CT-19 from 5 m away. Soon after, SS left there. Flies began to swarm the corpse.

**15:46** – BY descended to the ground and approached CT-19. HG was watching BY from a tree 2 m from BY. BY touched CT-19’s right arm for a few seconds, and he directed a threat face at the body. BY touched CT-19 again and sniffed it. Then, CT came running to HG and looked at BY, and she threat faced BY (Appendix 4).

**15:47** – BY touched CT-19’s hand and a protruding spinal bone, and he moved CT-19. CT exhibited a double threat with HG toward BY and CT-19.

**15:48** – BY touched CT-19’s spinal bone again and moved the corpse. Then, CT screamed and threated BY who quickly moved away from CT-19, though CT continued threating him. CT also threated HG.

**15:50** – HG and BY left the area. CT looked at CT-19 from 3 m away.

**15:52** – CT approached CT-19 and emitted an alarm call and trill calls toward CT-19, and she swatted away the flies that swarmed around the body. CT emitted an alarm call and moved away from CT-19, and directed a threat face toward the body from 5 m away into a tree.

**15:53** – CT and CE directed a double threat toward CT-19. Soon after, CT left and CE descended on the ground and approached CT-19. CE emitted trill calls and looked closely at CT-19.

**15:55** – CE touched CT-19 and emitted trill calls again, and she swatted flies away.

**15:56** – CE ascended a tree 2 m away and emitted an alarm call and threat face toward CT-19, and she moved 2 m higher in the tree while continuing to alarm call and threat face at the corpse.

**15:57** – At this time all group members left the area, moving out to forage.

**16:12** – No monkeys were returned to the area, and the observer collected the remains of CT-19 for photographs.

Altmann, J. (1974) ‘Observational study of behavior: sampling methods’, *Behaviour*, 49(3–4), pp. 227–266. doi: 10.1163/156853974X00534.
